# Analysis of lncRNA Expression in Patients With Eosinophilic and Neutrophilic Asthma Focusing on LNC_000127

**DOI:** 10.3389/fgene.2019.00141

**Published:** 2019-03-19

**Authors:** Yujin Zhu, Dan Mao, Wei Gao, Guojing Han, Hong Hu

**Affiliations:** ^1^Respiratory Department, Chinese People's Liberation Army General Hospital, Beijing, China; ^2^Respiratory Department, Tianjin Municipal Corps Hospital of CAPF, Tianjin, China; ^3^No. 968 Hospital of Chinese People's Liberation Army, Jinzhou, China

**Keywords:** long non-coding RNA, eosinophilic asthma, neutrophilic asthma, peripheral whole blood, RNA-sequencing

## Abstract

Long non-coding RNA (lncRNA) is important in many diseases. Some studies have shown that lncRNA affects the pathogenesis of systemic inflammation of asthma. lncRNA regulates gene transcription, protein expression, and epigenetic regulation. However, lncRNAs associated with different airway phenotypes, such as eosinophilic (Eos) and neutrophilic (Neu) asthma have not been identified. The goal of this study was to determine the differences in circulating lncRNA signatures in Eos and Neu samples. Using RNA-sequencing (RNA-seq), lncRNA expression was evaluated in peripheral whole blood samples among Eos patients, Neu patients, and healthy individuals (Control). Bioinformatic analysis was used to predict relevant biological pathways. Quantitative PCR (qPCR) was used to measure gene expression in whole blood samples, Jurkat cells, and human CD4^+^ T cells. Finally, a novel lncRNA, LNC_000127, was inhibited by transfection of Jurkat cells with a lentiviral vector, and the effect was examined by Human Asthma RT^2^ Profiler™ PCR Array and western blotting. Compared to control samples, Eos samples contained 190 unique lncRNAs and Neu samples had 166 unique lncRNAs (difference ≥2-fold). KEGG pathway annotation data and GO terms revealed that different lncRNAs are involved in different mechanisms. LNC_000127, was highly expressed in Eos samples before treatment; its expression was increased in Jurkat cells and human CD4^+^ T cells following stimulation with PMA/CD_28_. Subsequent analyses revealed that LNC_000127 functions in the Th_2_ inflammation pathway. The results suggest that lncRNAs are involved in different phenotypes of asthma. Whether the different phenotypes of asthma can be recognized based on these lncRNAs (as biomarkers) requires further analysis. Targeting LNC_000127 may be effective for reducing Th2 inflammation in Eos asthma.

## Introduction

### Background

According to the proportion of granulocytes in induced sputum, airway inflammation in asthma can be divided into four inflammatory phenotypes: eosinophilic (Eos), neutrophilic (Neu), paucigranulocytic, and mixed Eos/Neu asthma (Simpson et al., 2006). CD4^+^ T-helper cells can mainly be divided into four subsets, Th_1_/Th_2_/Th_17_/Treg, and the Th_2_ subset exerts critical effects on Eos asthma (Muehling et al., 2017; Shinoda et al., 2017). The key cytokines in the Th2 response include interleukin (IL)-4, IL-5, and IL-13 (Wynn, 2015). Some scholars have also attributed the incidence of asthma to a Th_2_/Treg imbalance (Chen et al., 2017). The driving mechanism of Neu asthma is associated with Th_1_/Th_17_ cells (Li et al., 2016; Chang et al., 2017). Gene expression analysis of Neu sputum and blood samples confirmed high expression of genes related to neutrophilic protease and α-defensins (Baines et al., 2011). Severe asthma is defined as partial or total unresponsiveness to asthma treatments and is always accompanied by an increase in eosinophil or neutrophil granulocytes (Chung et al., 2014). To improve asthma management, studies are needed to evaluate disease heterogeneity and develop tools for phenotype recognition to guide individualized treatment methods.

Conceptually, lncRNAs are transcripts longer than 200 bases that do not have coding potential and thus do not produce functional proteins (Zhang et al., 2009; Song et al., 2012). The use of next-generation sequencing of RNA (RNA-Seq) has demonstrated that the number of lncRNAs is much greater than the number of genes that encode proteins. However, recent studies suggested that a few lncRNAs with short open reading frames code for peptides with biological functions (Anderson et al., 2015; Matsumoto et al., 2017). The human genome contains ~51,382 lncRNA genes (Volders et al., 2015). LncRNAs may interact with other molecules, such as RNA (Du et al., 2016; Hu et al., 2016), DNA (Beckedorff et al., 2013; Villegas and Zaphiropoulos, 2015), proteins (Kotake et al., 2011; Warburton and Boone, 2017), and metal ions (Nelson et al., 2016). LncRNAs show great diversity in the human genome (Quinn and Chang, 2016). According to their genomic locations and manner of expression, lncRNAs can be categorized as intergenic, intronic, bidirectional, enhancer, sense, or antisense (Han et al., 2014; Xie et al., 2014). Dysregulation of lncRNA can lead to numerous diseases, including cancer, cardiovascular disease, diabetes, and some other complex disorders (Reddy et al., 2014; Wang et al., 2015; Shan et al., 2017). Recent studies have highlighted the emerging role of lncRNA in asthma (Perry et al., 2014). lncRNAs show differential expression in T cell development and differentiation (Hu et al., 2013; Kanduri et al., 2015) and lncRNA functions in regulating the development of Treg cells and dendritic cells (Qiao et al., 2013; Wang et al., 2014) or activated CD4^+^ T-cells (Xia et al., 2014). It is unclear whether a relationship exists between lncRNA and different asthma phenotypes.

### Objective

We evaluated the differences in circulating lncRNA signatures in Eos and Neu samples to determine whether lncRNAs are involved in Eos or Neu inflammation. Clustering analysis of differentially expressed genes (DEGs) was used for Eos vs. Neu samples. We characterized lncRNAs and predicted their functions by Gene Ontology (GO) analysis and Kyoto Encyclopedia of Genes and Genomes (KEGG) analysis using the mRNA-lncRNA co-expression network. In subsequent studies, we will examine the function of a novel lncRNA, LNC_000127, in the Th2 inflammation pathway.

## Methods

### Participants

Patients with onset Eos asthma (*n* = 12) and onset Neu asthma (*n* = 6) were selected according to the accepted standard (Eos asthma: induced sputum eosinophil count >3% and neutrophils <63%; Neu asthma: induced sputum eosinophil count <3% and neutrophils >63%) (Simpson et al., 2006). Exclusion criteria included recent (within the past weeks) respiratory tract infection, current smoking (or a history of smoking, within 6 months of cessation), and changes in maintenance therapy. All participants were selected from the People's Liberation Army General Hospital, and all participants provided informed consent before participating in the study. Healthy individuals were selected as control samples (*n* = 6). Clinical details for individual samples are shown in Table 1. The Ethics Committee of the People's Liberation Army General Hospital approved this study (Permit Number: S2016-089-01). Both informed and written consent was obtained from the participants.

**Table 1:** Main clinical and laboratory features of the asthma and control samples.

	**Eos**	**Neu**	**Control**	***p*-Value**
*N*	12	6	6	
Mean (SD) age, years	44 (11)	56 (12)	39 (12)	0.045
Gender, M/F	5/7	4/2	5/1	0.214
Atopy no. %	7 (58)	2 (33)	0 (0)	0.053
Heredity no. %	11 (92)^*^^#^	3 (50)^*^	0 (0)	0.001
Mean (SD) FeV_1_ %	65 (26)^*^	60 (26)^*^	98 (4)	0.018
Mean (SD) FeV_1_/FVC %	59 (17)^*^	60 (21)^*^	88 (3)	0.007
Mean (SD) BMI kg/m^2^	29.5 (8.7)	28.3 (5.6)	22.8 (2.9)	0.177
Mean (SD) FeNO (ppb)	42 (23)^*^	25 (9)	21 (9)	0.049
Smoking, ex/never	5/7	4/2	2/4	0.47
ACQ score, median (Q1–Q3)	1.1 (0.8–1.7)	1.1 (0.8–1.5)	N/A	
Mean (SD) WBC (10^∧^9/L)	4.4 (2.7–4.8)^#^	10.8 (6.7–15.5)^*^	3.9 (1.9–5.4)	<0.001
Neutrophils %, median (Q1–Q3)	27.4 (18.3–38.8)^#^	76.1 (71.5–87)	28.4 (12.1–50.3)	<0.001
Eosinophils %, median (Q1–Q3)	11.9 (4.4–20.6)^*^^#^	0.8 (0.0–1.5)	0.3 (0.1–0.8)	<0.001
Mean (SD) induced sputum Neu	31.9 (14.1)^#^	68.8 (5.8)^*^^#^	21.7 (12.5)	<0.001
Mean (SD) induced sputum Eos	6.9 (6.1)^*^^#^	0.3 (0.4)	0.8 (0.7)	0.008

FEV_1_, forced expiratory volume in the first second; FeV_1_/FVC, forced expiratory volume in the first second/forced vital capacity; BMI, body mass index; FeNO, higher fractional exhaled nitric oxide; WBC, white blood cell; ACQ, asthma control questionnaire score.

* p < 0.05 vs. Control

# *p < 0.05 vs. Neu*.

### Acquisition and Processing of Sputum Induction

Hypertonic saline (3% or 4.5%) was used for sputum induction. In the respiratory laboratory, each participant is subjected to a fixed sputum induction time (15 min). Selected sputum (sputum portion separated from saliva) was dispersed using dithiothreitol for inflammatory cell counts. The suspension was filtered, and then cell viability and the total cell count of leukocytes were determined.

### Whole Genome Gene Expression Analysis (RNA Isolation, Library Preparation, Sequencing, and Data Analysis)

Four milliliters of peripheral whole blood were collected by venipuncture at asthma onset. Two milliliters were used for RNA-seq analysis. Total cellular RNA was isolated from peripheral blood samples with TRIzol reagent (Invitrogen, Carlsbad, CA, USA) and stored at −80°C. RNA was isolated using the RNeasy mini extraction kit (#A419, Qiagen, Hilden, Germany) according to the manufacturer's instructions. RNA purity was checked using the NanoPhotometer® spectrophotometer (Implen, München, Germany). RNA concentration was measured using a Qubit® RNA Assay Kit in a Qubit® 2.0 Fluorometer (Life Technologies, Carlsbad, CA, USA). RNA integrity was assessed using the RNA Nano 6000 Assay Kit of the Bioanalyzer 2100 system (Agilent Technologies, Santa Clara, CA, USA). A total of 3 μg of RNA per sample was used as input material for the RNA sample preparations. The Illumina Hiseq 2500 platform (San Diego, CA, USA) was used to measure gene expression (see the Supplementary Text 1 for specific methods of RNA-seq). We treated the peripheral blood with TRIzol reagent without removing red blood cells. Because the nucleus of red blood cells is degraded, it contains no RNA. Therefore, the RNA of peripheral blood mainly originates from leukocyte. To reduce statistical errors, we used the leukocyte counts to adjust for expression differences. The level of RNA does not reflect changes in cell proportions, but one of the purposes of this study was to identify biomarkers in different asthma phenotypes. Peripheral whole blood is easy to obtain and store and is excellent for biomarker development. So differentially expressed mRNAs and lncRNAs in leukocytes were compared between Eos and Neu samples, and cut-off points of 0.5-fold for downregulation and 2-fold for upregulation of lncRNA expression were used. Student's *t*-test was used for gene expression analysis and a *p*-value < 0.05 was considered as statistically significant.

### GO and KEGG Enrichment Analysis

We conducted *trans* and *cis* analysis to evaluate the correlation between lncRNAs and mRNA expression. *Cis* role (co-location) of target gene prediction: The *cis* role is exerted when lncRNA acts on neighboring target genes. We searched for coding genes 10/100 k upstream and downstream of lncRNA and then analyzed their functions. *Trans* role (co-expression) of target gene prediction: The trans role occurs when lncRNAs identify each other based on expression levels. In terms of the modes of function, both *cis*-and *trans*-regulatory activities have been described previously (Mercer and Mattick, 2013). As *cis*-regulators, lncRNAs exert their functions on neighboring genes on the same allele from which they are transcribed, displaying an allele-specific correlation in expression and perturbation. GO and KEGG analysis is conducted using mRNA sequences. Through *cis* and *trans* studies, we identified lncRNAs suitable for analysis. In fact, both GO analysis and KEGG analysis can suggest the mechanism in which an RNA is involved.

To predict the ontology of differentially expressed lncRNA, GO enrichment analysis was conducted using the GOseq R package. GO terms were considered significantly enriched for DEGs if the corrected *p*-value was <0.05.

KEGG (http://www.genome.jp/kegg/) is a database resource for understanding processes in biological systems and high-level functions at the organism, cell, and ecosystem levels. By using molecular datasets generated by RNA-seq and the network between lncRNA and mRNA, we used KOBAS software to analyze the possible functions of differentially expressed lncRNA in the pathways.

### Cell Isolation, Culture, and Stimulation

1. Primary human CD4^+^ T cells were isolated from buffy coats of healthy donors using MACS negative CD4^+^ purification technology (Miltenyi Biotech, Bergisch Gladbach, Germany), yielding an overall 95% pure CD4^+^ T cell population.
2. The Jurkat cell line was obtained from the respiratory laboratory of the People's Liberation Army General Hospital.
3. Human CD4^+^ T and Jurkat cells were cultured in RP1640 (Solarbio, Beijing, China, Cat: 31800-500) supplemented with 10% fetal bovine serum. Cells were cultured at concentrations of 1–2 × 10^5^ cells/mL at 37°C/5% CO_2_.
4. Human CD4^+^ T and Jurkat cells were stimulated for 8–24 h with a combination of anti-CD_3_ (1 μg/mL, BioLegend, San Diego, CA, USA)/phorbol 12-myristate 13-acetate (PMA) (10 ng/mL, Lot #SLBT8132, Sigma, St. Louis, MO, USA) or anti-CD_28_ (1 μg/mL, pericluster CD_28_, Cat: 302902, BioLegend)/PMA.

### Generation of Stable Jurkat Cell Lines With LNC_000127 Knockdown

To generate a construct expressing LNC_000127 short hairpin RNA (shRNA) and LNC_000127 shRNA, the targeting sequences were cloned into the pLKD-CMV-EGFP-2A-puro-U6-shRNA vector. The resulting construct was named as pLKD-CMV-G&PR-U6-shRNA. A Jurkat cell line with stably knocked down LNC_000127 was generated by transfecting the Jurkat cells with pLKD-CMV-G&PR-U6-shRNA, followed by selection culture in puromycin (2 μg/mL, Sigma) for 2 weeks (knockdown group, KD). As controls, the Jurkat cell line was transfected with the empty vector pLKD-CMV- EGFP-2A-puro-U6-shRNA and stable lines were established as described above (normal control group, NC/CV). The lentivirus was supported by Obio Technology (Shanghai) Corp., Ltd. (Shanghai, China) and the procedures were conducted using standard protocols.

### Lentivirus Transduction

Jurkat cells were seeded into 48-well plates at 1 × 10^5^/well in 250 μL medium and infected by adding the proper lentivirus according to the protocol. After 24 h of transduction, fresh medium was added, and the cells were cultured for another 2 days before treatment.

### Quantitative Real-Time PCR

#### Confirmation of lncRNA Expression in Blood at Asthma Onset

By using TRIzol reagent (Invitrogen), total cellular RNA was isolated from peripheral whole blood samples. cDNA was synthesized with the Takara PrimeScript™ RT Master Mix Kit (Shiga, Japan). Using KAPA SYBR Fast Universal (Kapa Biosystems, Wilmington, MA, USA), we performed qPCR to evaluate lncRNA expression. The ABI Prism 7500 sequence detection system was used to perform qPCR (Applied Biosystems, Foster City, CA, USA). Briefly, the reactions were performed in a mixture (20 μL) containing 8 μL H_2_O, 10 μL 2X SYBR-Green PCRMix (Kapa), 1 μL cDNA template, and 0.5 μL each of sense and antisense primers. A total of 3 lncRNAs were tested. Primer sequences are listed in Table 2. We used GAPDH as a housekeeping gene for normalization. The relative changes in lncRNA expression were calculated using the 2^−ΔΔ*Ct*^ method. When the Eos asthma patient was cured, another 2 mL of blood was collected for verification of specific genes by RT-PCR. Statistical analysis was conducted as described above.

**Table 2:** Primers used to validate gene expression.

**Name**	**Forward primer (5^**′**^−3^**′**^)**	**Reverse primer**
ENST00000620143.1 (RP11-408H1.3)	AGTCACTACACTGGTATCTT	TCATATACCTCTGAG AGCTT
LNC_003333 (XLOC_000127)	ACCACTAACAGAAATACCAC	TCCCTCTAGAACTAAG AGAT
ENST00000500949.6 (OIP5-AS1)	AGTTGATTATAGCTCCTCTT	AGATGTATTAACATGG CTGT
GATA3	GTCCTGTGCGAACTGTCAGA	TCGGTTTCTGGTCTGG ATGC
IFN-γ	GGCTGTTTCTGGCTGTTACTGC	GACTCCTTTTCCGCTT CCTGA
GAPDH	TGCCACTCAGAAGACTGTGG	TTCAGCTCTGGGATG ACCTT

*GAPDH, glyceraldehyde-3-phosphate dehydrogenase*.

#### Confirmation of Gene Expression in Jurkat Cells

Total RNA was isolated from Jurkat cells by using TRIzol reagent as described above. Before and after the stimulation with PMA/CD_28_, three lncRNAs were examined by qPCR. Statistical analysis was conducted as described above.

#### Confirmation of lncRNA Expression in Human CD4^+^ T Cells

This process was conducted as described above. Before and after stimulation with PMA/CD_28_ and PMA/CD_3_, LNC_000127, GATA3 and INF-r were examined by qPCR. Primer sequences are listed in Table 2. Statistical analysis was conducted as described above.

#### Human Asthma RT^2^ Profiler™ PCR Array

By using TRIzol reagent, total cellular RNA was isolated from Jurkat stable cell strains KD and NC/CV (see the Supplementary Text 2 for specific steps used in this section).

### Western Blot Analysis

In addition to NC/CV cells, two other stable cell strains, KD and NC/CV, were collected after stimulation with PMA/CD_28_ for 8 h. Total cellular proteins were extracted according to standard procedures and the protein concentration was determined using Bradford reagent (Bio-Rad, Hercules, CA, USA). The isolated proteins were separated by SDS-PAGE and electrotransferred to polyvinyl difluoride membranes. Non-specific binding was blocked by incubating the membrane in 0.1% of Tween-20 in phosphate-buffered saline and 5% of dried milk for 1 h at 20–25°C. First, antibodies against GATA3, CD40L, CCR8, STAT5A, and CRLF2 were added and incubation was carried out overnight at 4°C. Next, the appropriate peroxidase-conjugated secondary antibody was added and immunoreactive bands were detected by chemiluminescence. Band intensities were quantified with ImageQuant software. The relative abundance of the above genes was calculated as the ratio of the normalized densitometric values between three samples. Intergroup differences in densitometry data were calculated by the Mann-Whitney U test using SPSS version 15.0 software (SPSS, Inc., Chicago, IL, USA). A *p*-value <0.05 was considered to indicate a statistically significant difference.

### Statistical Analysis

SPSS v11.6 was used for statistical analyses. All data are expressed as the mean ± SD. Differences between groups were assessed by one-way analysis of variance (ANOVA) and the Student-Newman-Keuls multiple comparison post-test or Student's *t*-test.

## Results

The clinic characteristics of the study samples (Eos *n* = 12, Neu *n* = 6, and control *n* = 6) are presented in Table 1. Compared to control samples, both the Eos and Neu samples showed lower forced expiratory volumes in the first second (FEV_1_%) and forced expiratory volumes in the first second/forced vital capacity (FeV_1_/FVC%). Eos samples showed higher fractional exhaled nitric oxide (FeNO) and induced sputum and blood eosinophil numbers; particularly, heredity values were higher than in the control and Neu samples. Neu samples showed higher induced sputum and blood neutrophil numbers. The ratio of smoking history in Neu samples was higher than in Eos samples, although the difference was not significant.

### DGE Analysis of mRNA and lncRNA

**Figure 1:**
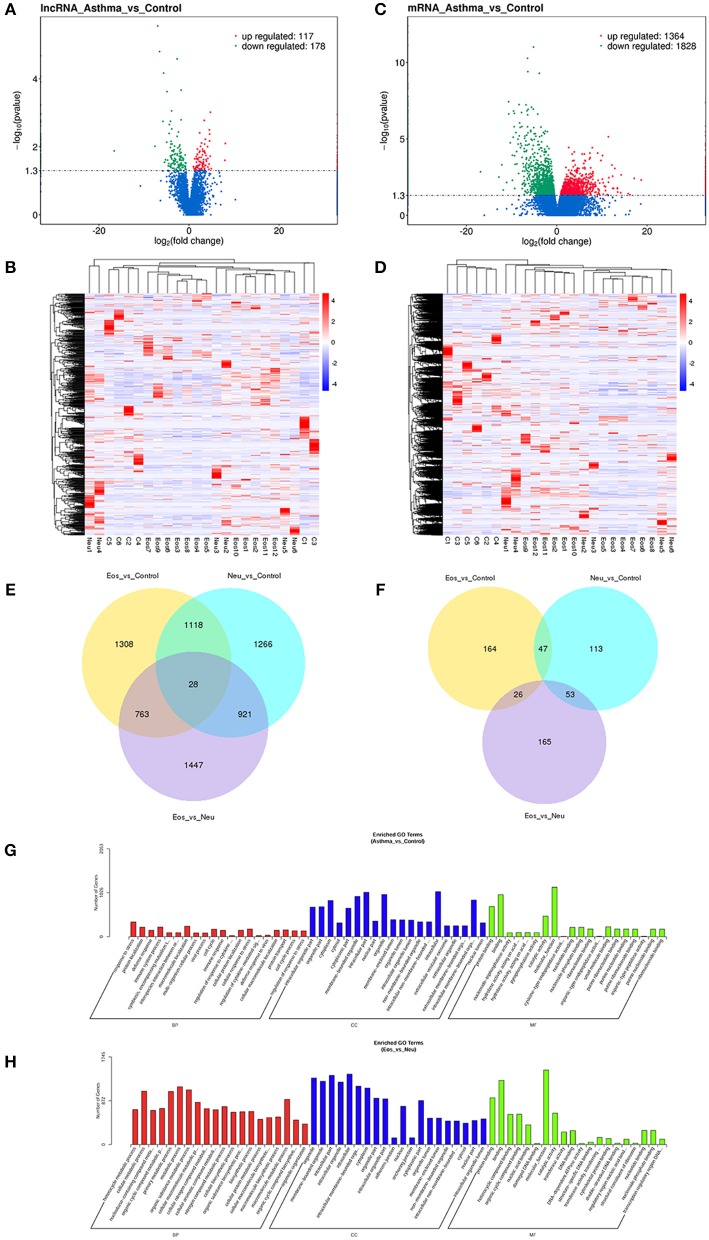
**(A)** Volcano plot assessment of lncRNA expression between asthma and control groups. Red spots indicate a >2.0-fold change in lncRNA expression between asthma and control groups. Green spots indicate a <0.5-fold change between asthma and control groups. **(B)** Heat map analysis of differentially expressed lncRNAs between asthma and control group. Blue indicates low lncRNA expression and red indicates high lncRNA expression. **(C)** Volcano plot assessment of mRNA expression between asthma and control groups. Red spots indicate a >2.0-fold change in mRNA expression between asthma and control groups. Green spots indicate a <0.5-fold change between asthma and control groups. **(D)** Heat map analysis of differentially expressed mRNAs between asthma and control group. Blue indicates low lncRNA expression and red indicates high lncRNA expression. **(E)** Venn diagram showing differential expression of mRNAs between asthma and control groups. **(F)** Venn diagram showing differential expression of lncRNA between asthma and control groups. **(G)** Gene Ontology (GO) analysis of differentially expressed lncRNAs between asthma and control groups. The most enriched GO terms targeted by dysregulated transcripts were involved in a variety of functions, such as immune response, immune system process, cellular response to stress, and response to stress. **(H)** Gene Ontology (GO) analysis of differentially expressed lncRNAs between Eos and Neu groups. The enriched GO term targeted by dysregulated transcripts was involved in multiple metabolic processes.

We analyzed the transcriptome of peripheral whole blood. The expression patterns differed significantly between asthma samples and control samples. Using 2-fold and 0.5-fold expression differences as cutoffs, a total of 295 lncRNA transcripts were specifically dysregulated (117 lncRNA transcripts upregulated and 178 lncRNA transcripts downregulated; each *p* < 0.05) in asthma samples compared to in control samples (Figures 1A,B). Additionally, a total of 3,192 mRNAs were specifically dysregulated, including 1,364 mRNAs upregulated and 1,828 mRNAs downregulated in asthma samples (Figures 1C,D).

We then performed differential expression analysis for all pair-wise comparisons: Eos vs. control samples, Neu vs. control samples, and Eos vs. Neu samples. In addition to the differences observed in gene expression between asthma and control samples, the Eos and Neu samples showed distinct gene expression profiles and clustered separately.

With respect to mRNA, a total of 6,851 mRNAs were significantly differentially expressed (*p-*value ≤0.05). Venn diagrams illustrating the overlap between the two differential expression analyses results are shown in Figure 1E. Each two sample sets showed distinct mRNA expression profiling patterns (see Supplementary Tables 1–4). Eos asthma is known to be associated with the Th2 pathway and IL4, IL5, and IL13 are involved in “Th2-high” asthma. In the present study, as shown in Supplementary Table 4 (mRNA_Eos_vs_Neu), some genes, such as IL4R (receptor of IL4, IL13) and IL5R were significantly upregulated in Eos samples.

Similarly, a total of 568 lncRNAs were differentially expressed (*p*-value ≤0.05). Venn diagrams illustrating the overlap between the two differential expression analyses results are shown in Figure 1F. Compared to control samples, Eos samples contained 190 unique lncRNAs and Neu samples had 166 unique lncRNAs (difference ≥2-fold). Each two sample sets showed distinct lncRNA expression profile patterns (see Supplementary Tables 5–8).

### GO term and KEGG Pathway Analysis of lncRNAs Differing Between Asthma and Control Samples

**Figure 2:**
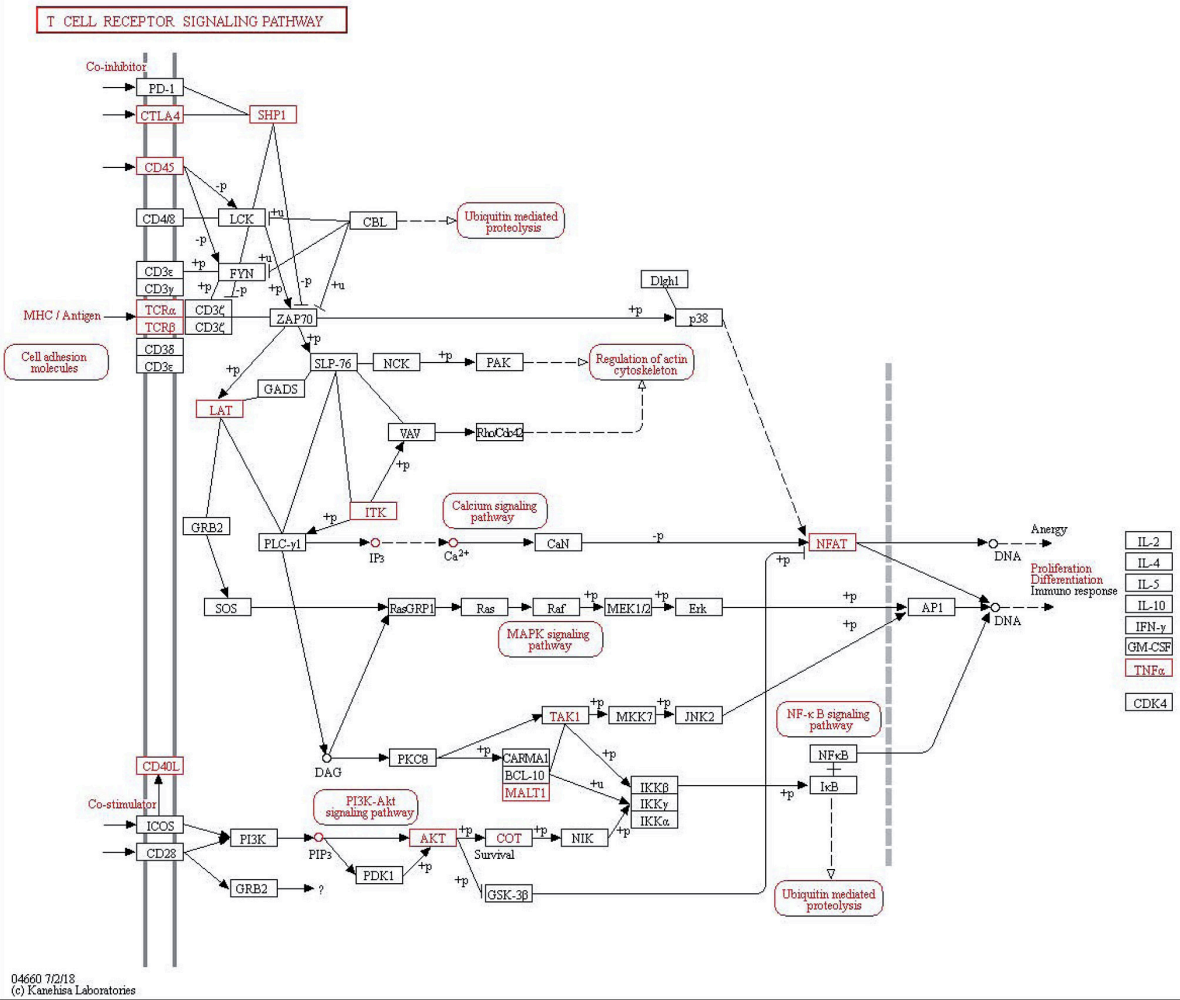
Kyoto Encyclopedia of Genes and Genomes (KEGG) pathway analysis of T cell receptor signaling pathway in asthma.

A comparison of asthma with control samples using GO analysis showed that upregulated and downregulated transcripts were involved in immune response, immune system process, cellular response to stress, and response to stress (Figure 1G). The most significant molecular function enrichment scores are also shown in Figure 1G. KEGG pathway annotation indicated that the most enriched pathways were measles, JAK-STAT signaling pathway, TNF signaling pathway, cytokine-cytokine receptor interaction (see Supplementary Table 9). As one of the most important pathways, the T cell receptor signaling pathway is listed in Figure 2.

### GO Term and KEGG Pathway Analysis of lncRNAs Differing Between Eos and Neu Samples

Comparison of Eos with Neu samples using GO analysis showed that upregulated and downregulated transcripts were involved in multiple metabolic processes (Figure 1H). KEGG pathway annotation indicated that the most enriched pathways were chemokine signaling pathway, B cell receptor signaling pathway, cytokine-cytokine receptor interaction, and tumor necrosis factor (TNF) signaling pathway (see Supplementary Table 10).

### Prediction of lncRNA Target Genes Associated With Eos Asthma

To determine the association of lncRNAs and directly regulated expression of target mRNAs, we carried out lncRNA target predictions depending on the mRNA-lncRNA correlation network. Pearson's correlation analysis was performed using a coefficient ≤0.95 to construct the network. We choose several genes associated with Eos asthma, including Il5RA, GATA3, SATAT5, and SOCS, to identify co-expressed lncRNAs. Among the dysregulated lncRNAs, 3 lncRNAs (RP11-408H1.3, LNC_000127, OIP5-AS1) were found to be co-expressed with the above genes.

### Confirmation of Dysregulated lncRNAs in Asthma vs. Control Samples

**Figure 3:**
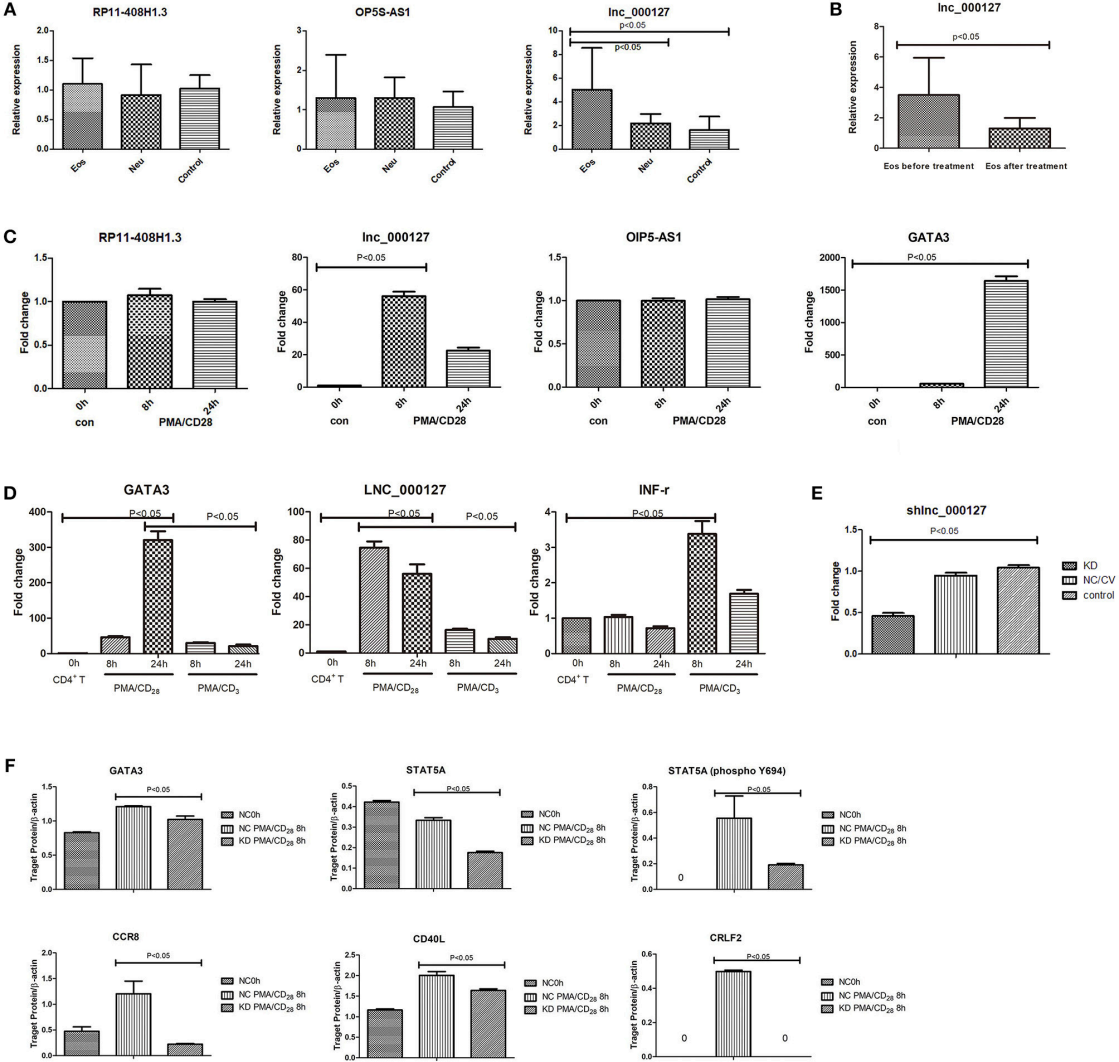
**(A)** Three selected lncRNA expression levels were validated *in vivo*. The expression of the selected three lncRNAs was validated by qPCR in three groups *in vivo*. LNC_000127 was upregulated in Eos samples (*p* < 0.05). **(B)** Before and after treatment, the expression of LNC_000127 was validated by qPCR in Eos samples *in vivo*. LNC_000127 was downregulated after treatment in Eos samples (*p* < 0.05). **(C)** lncRNA expression in response to PMA/CD_28_ stimulation. Jurkat cells were treated without (control) or with 10 ng/mL PMA, and 1 μg/mL CD_28_ for 8 and 24 h. Levels of selected lncRNAs and GATA3 were determined by qPCR, using the housekeeping gene GAPDH as a reference. Data are shown as the mean ± SD (*n* = 3) and are representative of one of three independent experiments. *p* < 0.05 compared to the control group, as analyzed by one-way ANOVA and *post-hoc* Bonferroni test. **(D)** Human CD4^+^ T cells were divided into three groups. One group was treated with 10 ng/mL PMA, and 1 μg/mL CD_28_ for 8 and 24 h. One group was treated with 10 ng/mL PMA, and 1 μg/mL CD_3_ for 8 and 24 h. The last group was used as a control. Gene expression results were validated by qPCR. Levels of selected genes (GATA3, LNC_000127, and INF-r) were determined by real-time PCR using the housekeeping gene GAPDH as a reference. Data are shown as the mean ± SD (*n* = 3) and are representative of one of three independent experiments. A *p*-value <0.05 compared to the control group, as analyzed by one-way ANOVA and *post-hoc* Bonferroni test. **(E)** Expression of LNC_000127 was validated by qPCR. Data are shown as the mean ± SD (*n* = 3) and are representative of one of three independent experiments. *p*-Value <0.05 compared to the control group, as analyzed by one-way ANOVA and *post-hoc* Bonferroni test. **(F)** Expression of the genes was validated by western blotting. Gene expression in response to PMA/CD_28_ stimulation. Jurkat cells were treated without (control) or with 10 ng/mL PMA, and 1 μg/mL CD_28_ for 8 h. β-Actin was used as a reference. The relative abundance of genes was calculated as the ratio of the normalized densitometric values between three samples. Intergroup differences (of the densitometry data) were calculated by the Mann-Whitney *U* test using SPSS version 11.6 software. *p*-Value <0.05 was considered to indicate a statistically significant difference.

To confirm the sequencing gene data, we further analyzed the above 3 dysregulated lncRNAs by qPCR. One lncRNA, LNC_000127, was identified because its expression showed the highest conformance and stability, which agreed with the sequencing results (Figure 3A). LNC_000127 showed significant differences in expression between the three groups *in vivo* and was upregulated in the Eos group. When the Eos asthma patient was cured, 2 mL of peripheral whole blood was collected for LNC_000127 verification *in vivo*. LNC_000127 expression showed a decreasing trend (*p* < 0.05) after treatment (Figure 3B).

### LNC_000127 Showed Altered Expression in Response to PMA/CD_28_ Stimulation

Eos asthma is closely related to the Th_2_ pathway and Neu asthma is correlated with the Th1 pathway. Translation toward primary purified human CD4^+^ T cells revealed that PMA/CD_3_ stimulation induced a more pronounced Th1-specific Interferon Gamma(INF-γ), whereas PMA/CD_28_ induced Th2-specific GATA3 production (Smeets et al., 2012). Differential stimulation observed in the Jurkat assay is applicable in the primary human cellular assay and depends on the same proximal signaling hubs (Smeets et al., 2012). Thus, we used Jurkat cells in this study. Of the above 3 lncRNAs, LNC_000127 showed the greatest increase in PMA/CD_28_-treated Jurkat cells. LNC_000127 was upregulated at 8 h after PMA/CD_28_ stimulation. Twenty-four hours later, a decreasing trend was observed. Additionally, the expression of GATA3 was already increased at 8 h PMA/CD_28_ treatment (Figure 3C). Human CD4^+^ T cells were used to validate the effects of specific genes after stimulation by PMA/CD_28_ and PMA/CD_3_. PMA/CD_3_ appeared to be most powerful stimulus inducing IFN-r, whereas PMA/CD_28_ induced GATA3 and LNC_000127 production. These data suggest that LNC_000127 affects the Th2 pathway rather than the Th1 pathway (Figure 3D). Therefore, we further examined the function of LNC_000127.

### Characterization of LNC_000127 Expression

1. Full-length LNC_000127 is ~1,561 nucleotides in length (sequence shown in Supplementary Text 3). LNC_000127 is located ~8,000 base pairs upstream of the known sequence of GBP6 (NCBI ENST00000370456) and is a novel lncRNA.
2. We determined the subcellular localization of LNC_000127. To do this, fluorescence *in situ* hybridization was conducted in Jurkat cells. We found that most LNC_000127 was present in the nuclei (Supplementary Image 1). These data suggest that LNC_000127 exerts its functions in the nucleus.
3. Through the experiments described above, we found that LNC_000127 expression was increased in the Th2 pathway in CD4^+^ T cells and Jurkat cells. Peripheral blood detection before and after treatment of Eos patients showed that LNC_000127 expression was decreased (*p* < 0.05). This suggests that LNC_000127 can be used as a biomarker for the onset and prognosis of patients. Studies with very large sample sizes are required to verify this result.

### LNC_000127 Regulates PMA/CD_28_-Induced Inflammatory Response

To determine the role of LNC_000127 in the PMA/CD_28_-induced inflammatory response, we used a knockdown strategy in Jurkat cells using a lentiviral system (KD group). As controls, the Jurkat cell line was transfected with empty vector (NC/CV group). To exclude the possibility that transient transfection had non-specific effects on cellular responses, we generated a cell line with stable knockdown of LNC_000127 and cell line with empty vector (Supplementary Image 2). As shown in Figure 3E, the level of LNC_000127 was downregulated in the KD group, indicating that the knockdown strategy was successful.

To determine the mechanisms by which LNC_000127 regulates the PMA/CD_28_-induced inflammatory response, the Human Asthma RT^2^ Profiler™ PCR Array was used. A total of 84 gene related to asthma were compared between the KD and NC/CV groups. Thirty-four genes were differentially expressed between the KD and NC/CV groups by ≥2-fold. A total of 20 genes were upregulated and 14 were downregulated (see Supplementary Table 11). The main dysregulated genes were interleukins, chemokines, and T cell surface receptors.

The expression of some genes (GATA3, CD40L, CCR8, STAT5A, and CRLF2) after stimulation by PMA/CD_28_ was significantly decreased in the KD group according to our PCR array analysis. Decreased protein expression was confirmed by western blot analysis (Figure 3F and Supplementary Image 3). The results indicate that the average levels of genes (GATA3, CD40L, CCR8, STAT5A, and CRLF2) in the NC/CV group were significantly higher than those in the KD group (*p* < 0.05).

## Discussion

lncRNAs can be categorized as intergenic, intronic, bidirectional, enhancer, sense, or antisense. Combining the epigenetic function with the variability, plasticity, and tissue specificity, lncRNAs are regarded as key regulators of disease. No studies have examined lncRNA expression in eosinophilic and neutrophilic asthma. In this study, compared to in control samples, Eos samples contained 190 unique lncRNAs and Neu samples contained 166 unique lncRNAs (difference ≥2-fold). Some of these differentially expressed lncRNAs may be involved in the chemokine signaling pathway, B cell receptor signaling pathway, cytokine-cytokine receptor interaction, and TNF signaling pathway. We demonstrated that LNC_000127 was upregulated in Eos samples by qPCR and was downregulated after treatment. LNC_000127, which is located upstream of the GBP6 transcript, may participate in and contribute to eosinophilic asthma.

In the present study, we found that the expression of LNC_000127 was increased in response to PMA/CD_28_ stimulation in CD4^+^ T cells and Jurkat cells. This suggests that LNC_000127 is involved in regulated the Th2 inflammatory response. To characterize the role of LNC_000127 in this process, we used a knockdown strategy in Jurkat cells using a lentiviral system. GATA3 and STAT5A can be induced by PMA/CD_28_, whereas knockdown of LNC_000127 resulted in significant inhibition of both GATA3 and STAT5A. In response to PMA/CD_28_, we found that knockdown of LNC_000127 decreased the expression of CCR8, CRLF2, and CD40L (Th2 inflammatory receptors). This suggests that LNC_000127 is a positive regulator of PMA/CD_28_-induced Th2 inflammation. Such findings in Jurkat cells are particularly important because a dysregulated immune response in T cell is closely associated with the development of Eos asthma.

lncRNAs have been reported to exhibit distinct profiles in immune processes. Airway smooth muscle cells contribute to asthma via airway remodeling. The lncRNA BCYRN1 similarly increased airway smooth muscle proliferation and migration in an asthma model (Zhang et al., 2016). Another study showed that expression of the lncRNA MEG3 was reduced in circulating CD8^+^ T cells in patients with severe asthma, in addition to 18 other lncRNAs (Tsitsiou et al., 2012). For glucocorticoid-resistant asthma, lncRNA GAS5 was found to function as a glucocorticoid receptor (Kino et al., 2010). Pro-inflammatory mediators up-regulate GAS5 levels in both smooth muscle cells and the airway epithelia (Keenan et al., 2015). An *in vivo* study supported the role of lncRNA in therapy-resistant asthma. The expression of NEAT1 and PINT (AC058791.2) is upregulated in children with therapy-resistant asthma (Persson et al., 2015). lncRNAs expressed from intergenic transcripts were also shown to have a supporting function in Th2 cell differentiation (Hu et al., 2013; Kanduri et al., 2015). A whole-genome sequencing approach identified differently lncRNAs to be expressed in human Th1, Th2, and Th17 cells (Spurlock et al., 2015). The Th2 locus control region (Th2-LCR) upregulated the expression of IL-5, IL-4, and IL-13. TH2-LCR showed Th2-specific demethylation after antigenic stimulation (Koh et al., 2010). Thus, TH2-LCR is critically important in Th2 responses *in vivo* and *in vitro* (Spurlock et al., 2015). A previous study showed that LINC00882, LINC00883, and PVT1 are differentially expressed in the primary airway smooth muscle cell phenotype (Perry et al., 2014). PVT1 is decreased in patients with corticosteroid-sensitive non-severe asthma and increased in patients with corticosteroid-insensitive severe asthma (Austin et al., 2017). Thus, PVT1 may be a therapeutic target for reducing airway remodeling (Yu et al., 2017). Our results revealed that LNC_000127 participates in PMA/CD_28_-induced cellular activation, suggesting that inflammation is regulated not only by small non-coding RNAs and protein modulators, but also by lncRNA. Particularly, the involvement of LNC_000127 appears to add another layer of complexity to regulation of the inflammatory response. Th2 differentiation occurs primarily via the JAK-STAT signaling pathway and STAT5A is a major STAT in the JAK-STAT pathway. Our results suggest that LNC_000127 plays roles in the TCR/STAT/GATA3 pathway in the Th2 inflammatory response.

## Conclusion

Because Eos and Neu asthma have different inflammatory phenotypes, it is useful to analyze gene expression to explore their pathogenesis. Our results provide insight into the peripheral whole blood transcriptome lncRNA. Peripheral whole blood is easy to obtain and store and thus is an excellent source of biomarkers. Whether LNC_000127 can be used as a new therapeutic target and diagnostic biomarker for Eos asthma must be confirmed in further studies.

## Data Availability

The sequencing data have been submitted to GEO and are accessible through the accession numbers GSE106230 and GSE117038.

## Author Contributions

HH conceived and designed the study and revised the manuscript. YZ performed the RNA-related experiments and data analysis and drafted the manuscript. DM and WG participated in the experimental design and sample collection. GH conducted qPCR validation. All authors read and approved the final manuscript.

### Conflict of Interest Statement

The authors declare that the research was conducted in the absence of any commercial or financial relationships that could be construed as a potential conflict of interest.

## Supplementary Material

Supplementary Text 1 Text that presents detailed information on data analysis.

Supplementary Text 2 Method of PCR Assay SABio96.

Supplementary Text 3 Sequence of LNC_000127.

Supplementary Image 1 FISH experiment. Most lnc_000127 was detected in the nuclei. Green for the target gene, blue for DAPI, and third for composite image.

Supplementary Image 2 Three sets of white light and fluorescent photos. **(A,B)** KD group; **(C,D)** NC group; **(E,F)** Control group. Lentivirus transfection was successful in the KD and NC groups.

Supplementary Image 3 Western blotting. The decrease in protein (GATA3, CD40L, CCR8, STAT5A, and CRLF2) expression was confirmed by western blot analysis in the KD and NC/CV groups.

Supplementary Tables 1–4 showing significant mRNA annotation.

Supplementary Tables 5–8 showing significant lncRNA annotation.

Supplementary Table 9 presents KEGG pathway analysis for lncRNAs that differed between asthma and control samples.

Supplementary Table 10 presents KEGG pathway analysis for lncRNAs that differed between Eos and Neu samples.

Supplementary Table 11 presents the results of PCR Assay SABio96s (KD vs. NC/CV).

## Abbreviations

lncRNA: long non-coding RNA
mRNA: messenger RNA
Eos: eosinophilic asthma
Neu: neutrophilic asthma
GO: gene ontology
KEGG: Kyoto encyclopedia of genes and genomes
RT-PCR: real-time polymerase chain reaction
NC/CV: normal control group
KD: knockdown group
TNF: tumor necrosis factor
FISH: fluorescence *in situ* hybridization
GAPDH: glyceraldehyde-3-phosphate dehydrogenase
Th2: T-helper cell type 2
ILC2: innate lymphoid cell type 2
DEGs: differentially expressed genes
FEV_1_: forced expiratory volume in the first second
FeNO: higher fractional exhaled nitric oxide
MF: molecular function.
