# Supplementary material for: Analysis of lncRNA Expression in Patients With Eosinophilic and Neutrophilic Asthma Focusing on LNC_000127
Source: Front Genet. 2019 Mar 19;10:141. doi: 10.3389/fgene.2019.00141 (PMC6433975; doi:10.3389/fgene.2019.00141)
Supplement: Supplementary Text 1 — Text that presents detailed information on data analysis. [file Data_Sheet_1.zip › Supplementary Image1-FISH experiment.doc]

Supplemental Image1- FISH experiment.

Most lnc_000127 was detected in the nuclei. Green for the target gene, blue for DAPI, and third for composite image.
