# Supplementary material for: Analysis of lncRNA Expression in Patients With Eosinophilic and Neutrophilic Asthma Focusing on LNC_000127
Source: Front Genet. 2019 Mar 19;10:141. doi: 10.3389/fgene.2019.00141 (PMC6433975; doi:10.3389/fgene.2019.00141)
Supplement: Supplementary Text 1 — Text that presents detailed information on data analysis. [file Data_Sheet_1.zip › Supplementary Image2-Three sets of white light and fluorescent photos.doc]

Supplemental Image2- Three sets of white light and fluorescent photos.

a–b, KD group; c–d, NC group; e–f, Control group. Lentivirus transfection was successful in the KD and NC groups.
