# Supplementary material for: Analysis of lncRNA Expression in Patients With Eosinophilic and Neutrophilic Asthma Focusing on LNC_000127
Source: Front Genet. 2019 Mar 19;10:141. doi: 10.3389/fgene.2019.00141 (PMC6433975; doi:10.3389/fgene.2019.00141)
Supplement: Supplementary Text 1 — Text that presents detailed information on data analysis. [file Data_Sheet_1.zip › Supplementary Image3-Western blotting.doc]

Supplemental Image3- Western blotting

The decrease in protein (GATA3, CD40L, CCR8, STAT5A, and CRLF2) expression was confirmed by western blot analysis in the KD and NC/CV groups.
