# Supplementary material for: Analysis of lncRNA Expression in Patients With Eosinophilic and Neutrophilic Asthma Focusing on LNC_000127
Source: Front Genet. 2019 Mar 19;10:141. doi: 10.3389/fgene.2019.00141 (PMC6433975; doi:10.3389/fgene.2019.00141)
Supplement: Supplementary Text 1 — Text that presents detailed information on data analysis. [file Data_Sheet_1.zip › Supplementary Text1-The methods of RNA-seq.doc]

***The methods of RNA-seq***

***Library preparation for lncRNA sequencing***

A total of 3 μg of RNA per sample was used as input material for the RNA sample preparations. Firstly, ribosomal RNA was isolated using the Epicentre Ribo-zero™ rRNA Removal Kit (Epicentre, USA), and the rRNA-free material was cleaned by ethanol precipitation. Subsequently, sequencing libraries were generated using the rRNA-depleted RNA using a NEBNext® Ultra™ Directional RNA Library Prep Kit for Illumina® (NEB, USA) following the manufacturer’s recommendations. Briefly, fragmentation was carried out using divalent cations under elevated temperature in NEBNext First Strand Synthesis Reaction Buffer (5X). First strand cDNA was synthesized using a random hexamer primer and M-MuLV Reverse Transcriptase (RNaseH-). Second strand cDNA synthesis was subsequently performed using DNA Polymerase I and RNase H. In the reaction buffer, dTTPs were replaced by dUTP. Remaining overhangs were converted into blunt ends via exonuclease/polymerase activities. After adenylation of the 3ʹ ends of the DNA fragments, a NEBNext Adaptor with a hairpin loop structure was ligated in preparation for hybridization. In order to select cDNA fragments of 150–200 bp in length, the library fragments were purified with the AMPure XP system (Beckman Coulter, Beverly, USA). Then 3 μl of USER Enzyme (NEB, USA) was used with size-selected, adaptor-ligated cDNA at 37°C for 15 min followed by 5 min at 95°C before PCR. PCR was performed with Phusion High-Fidelity DNA polymerase, Universal PCR primers, and Index (X) Primer. At last, products were purified (AMPure XP system) and library quality was assessed on the Agilent Bioanalyzer 2100 system.

***Clustering and sequencing***

The clustering of the index-coded samples was performed on a cBot Cluster Generation System using a TruSeq PE Cluster Kit v3-cBot-HS (Illumina) according to the manufacturer’s instructions. After cluster generation, the libraries were sequenced on an Illumina Hiseq 2500 platform and 125-bp paired-end reads were generated.

***Data analysis***

Quality control: Raw data (raw reads) in fastq format were processed using in-house perl scripts. Clean data (clean reads) were obtained by removing reads containing adapter sequence, poly-Ns, and low-quality reads. At the same time, the Q20, Q30, and GC content of the clean data were calculated. All downstream analyses were performed using this high-quality clean data.

Mapping to the reference genome: Reference genome and gene model annotation files were downloaded from the genome website directly. The index of the reference genome was built using Bowtie v2.0.6 and paired-end clean reads were aligned to the reference genome using TopHat v2.0.9 (see the Supplemental Content).

***Transcriptome assembly***: The mapped reads of each sample were assembled by both Scripture (beta2) (**Guttman et al. 2010**) and Cufflinks (v2.1.1) (**Trapnell et al. 2010**) in a reference-based approach. Both methods use spliced reads to determine exons connectivity, but with two different approaches. Scripture uses a statistical segmentation model to distinguish expressed loci from experimental noise and uses spliced reads to assemble expressed segments. It reports all statistically expressed isoforms in a given locus. Cufflinks uses a probabilistic model to simultaneously assemble and quantify the expression level of a minimal set of isoforms that provides a maximum likelihood explanation of the expression data in a given locus. Scripture was run with default parameters, Cufflinks was run with ‘min-frags-per-transfrag=0’ and ‘--library-type’, other parameters were set as default.

***Coding potential analysis:*** CNCI (Coding-Non-Coding-Index) (v2) profiles adjoining nucleotide triplets to effectively distinguish protein-coding and non-coding sequences independent of known annotations (**Sun et al. 2013**). We use CNCI with default parameters. CPC (Coding Potential Calculator) (0.9-r2) mainly through assess the extent and quality of the ORF in a transcript and search the sequences with known protein sequence database to clarify the coding and non-coding transcripts (**Kong et al. 2007**). We used the NCBI eukaryotes' protein database and set the e-value ‘1e-10’ in our analysis. Pfam-scan We translated each transcript in all three possible frames and used Pfam Scan (v1.3) to identify occurrence of any of the known protein family domains documented in the Pfam database (release 27; used both Pfam A and Pfam B) （**Punta, et al. 2012**） . Any transcript with a Pfam hit would be excluded in following steps. Pfam searches use default parameters of -E 0.001 --domE 0.001 (**Bateman, et al. 2002**).

PhyloCSF (phylogenetic codon substitution frequency) (v20121028) examines evolutionary signatures characteristic to alignments of conserved coding regions, such as the high frequencies of synonymous codon substitutions and conservative amino acid substitutions, and the low frequencies of other missense and non-sense substitutions to distinguish protein-coding and non-coding transcripts **(Lin et al. 2011)**. We build multi-species genome sequence alignments and run phyloCSF with default parameters. Transcripts predicted with coding potential by either/all of the four tools above were filtered out, and those without coding potential were our candidate set of lncRNAs.

***Conservative analysis***

Phast (v1.3) is a software package contains much of statistical programs, most used in phylogenetic analysis (**Siepel, et al. 2005**), and phastCons is a conservation scoring and identificating program of conserved elements. We used phyloFit to compute phylogenetic models for conserved and non-conserved regions among species and then gave the model and HMM transition parameters to phyloP to compute a set of conservation scores of lncRNA and coding genes.

***Target gene prediction*** ²

Cis role (Co-location) of target gene prediction: Cis role is lncRNA acting on neighboring target genes. We searched coding genes 10k/100k upstream and downstream of lncRNA and then analyzed their function next.

Trans role (Co-expression) of target gene prediction: Trans role is lncRNA to identify each other by the expression level. While there were no more than 25 samples, we calculated the expressed correlation between lncRNAs and coding genes with custom scripts; otherwise, we clustered the genes from different samples with WGCNA (**Langfelder et al, 2008**) to search common expression modules and then analyzed their function through functional enrichment analysis.

***Quantification of gene expression level***

Cuffdiff (v2.1.1) was used to calculate FPKMs of both lncRNAs and coding genes in each sample (**Trapnell, C. et al. 2010**). Gene FPKMs were computed by summing the FPKMs of transcripts in each gene group. FPKM means fragments per kilo-base of exon per million fragments mapped, calculated based on the length of the fragments and reads count mapped to this fragment

***Differential expression analysis***

The Ballgown suite includes functions for interactive exploration of the transcriptome assembly, visualization of transcript structures and feature-specific abundances for each locus, and post-hoc annotation of assembled features to annotated features(Alyssa C. Frazee et al.2014). Transcripts with an P-adjust <0.05 were assigned as differentially expressed. Cuffdiff provides statistical routines for determining differential expression in digital transcript or gene expression data using a model based on the negative binomial distribution **(Trapnell, C. et al. 2010)**. Transcripts with an P-adjust <0.05 were assigned as differentially expressed.

Guttman, M., et al. (2010). Ab initio reconstruction of cell type-specific transcriptomes in mouse reveals the conserved multi-exonic structure of lincRNAs. Nature biotechnology.(scripture)

Trapnell, C. et al. (2010).Transcript assembly and quantification by RNA-seq reveals unannotated transcripts and isoform switching during cell differentiation. Nat. Biotechnol.(Cufflinks)、

Sun L, Luo H, Bu D, et al. (2013). Utilizing sequence intrinsic composition to classify protein-coding and long non-coding transcripts. Nucleic acids research, 41(17): e166-e166.(CNCI)

Kong, L., Y. Zhang, Z.Q. Ye, X.Q. Liu, S.Q. Zhao, L. Wei, and G. Gao. (2007). CPC: assess the protein-coding potential of transcripts using sequence features and support vector machine. Nucleic Acids Res 36: W345-349. (CPC)

M. Punta, P.C. Coggill, R.Y. Eberhardt, et al. (2012). The Pfam protein families database: Nucleic Acids Research , Database Issue 40:D290-D301. (pfam scan)

Bateman A, Birney E, Cerruti L, et al. (2002). The Pfam protein families database. Nucleic acids research, 30(1): 276-280.(pfam-scan)

Lin M F, Jungreis I, Kellis M. (2011). PhyloCSF: a comparative genomics method to distinguish protein coding and non-coding regions. Bioinformatics, 27(13): i275-i282.（phyloCSF）

Siepel, A., Bejerano, G., Pedersen, J.S., et al. (2005). Evolutionarily conserved elements in vertebrate, insect, worm, and yeast genomes. Genome Res. 15, 1034-1050. (Phast)

Langfelder, P.,Horvath, S. (2008). WGCNA: an R package for weighted correlation network analysis. (coexpression)

Young, M. D., Wakefield, M. J., Smyth, G. K., and Oshlack, A. (2010).Gene ontology analysis for RNA-seq: accounting for selection bias. Genome Biology. (GOseq)
