# Supplementary material for: Analysis of lncRNA Expression in Patients With Eosinophilic and Neutrophilic Asthma Focusing on LNC_000127
Source: Front Genet. 2019 Mar 19;10:141. doi: 10.3389/fgene.2019.00141 (PMC6433975; doi:10.3389/fgene.2019.00141)
Supplement: Supplementary Text 1 — Text that presents detailed information on data analysis. [file Data_Sheet_1.zip › Supplementary Text2-PCR Assay SABio96.doc]

# RT^2^ RNA QC PCR Arrays Protocol

# RNA ISOLATION

- **ASSESING RNA YIELD AND QUALITY**
- **DNase treatment**
- **RNA cleanup**
- **First Strand cDNA Synthesis**
- **Performing Real-Time PCR**
- **Data Analysis**

# RNA ISOLATION

Reagent:

TRIZOL® Reagent (Invitrogen)

Chloroform

Isopropyl alcohol

75% Ethanol (in DEPC-treated water)

RNase-free water

RNase-free glycogen (Ambion)

**1. HOMOGENIZATION**

a. *Tissues*

Homogenize tissue samples in 1 ml of TRIZOL Reagent per 50-100 mg of tissue using a power homogenizer. The sample volume should not exceed 10% of the volume of TRIZOL Reagent used for homogenization.

b. *Cells Grown in Monolayer*

Lyse cells directly in a culture dish by adding 1 ml of TRIZOL Reagent to a 3.5 cm diameter dish, and passing the cell lysate several times through a pipette. The amount of TRIZOL Reagent added is based on the area of the culture dish (1 ml per 10 cm2) and not on the number of cells present.

c. *Cells Grown in Suspension*

Pellet cells by centrifugation. Lyse cells in TRIZOL Reagent by repetitive pipetting. Use 1 ml of the reagent per 5-10 × 10^6^ of animal, plant or yeast cells, or per 1 × 10^7^ bacterial cells. Washing cells before addition of TRIZOL Reagent should be avoided as this increases the possibility of mRNA degradation. Disruption of some yeast and bacterial cells may require the use of a homogenizer.

**2. PHASE SEPARATION**

Incubate the homogenized samples for 5 minutes at 15 to 30°C to permit the complete dissociation of nucleoprotein complexes. Add 0.2 ml of chloroform per 1 ml of TRIZOL Reagent. Cap sample tubes securely. Shake tubes vigorously by hand for 15 seconds and incubate them at 15 to 30°C for 2 to 3 minutes. Centrifuge the samples at 12,000 × *g* for 15 minutes at 4°C. Following centrifugation, the mixture separates into a lower red, phenol-chloroform phase, an interphase, and a colorless upper aqueous phase. RNA remains exclusively in the aqueous phase. The volume of the aqueous phase is about 60% of the volume of TRIZOL Reagent used for homogenization.

**3. RNA PRECIPITATION**

Transfer the aqueous phase to a fresh tube. Precipitate the RNA from the aqueous phase by mixing with isopropyl alcohol. Use 0.5 ml of isopropyl alcohol per 1 ml of TRIZOL Reagent used for the initial homogenization. Incubate samples at 15 to 30°C for 10 minutes and centrifuge at 12,000 × *g* for 10 minutes at 4°C. The RNA precipitate, often invisible before centrifugation, forms a gel-like pellet on the side and bottom of the tube.

**4. RNA WASH**

Remove the supernatant. Wash the RNA pellet once with 75% ethanol, adding at least 1 ml of 75% ethanol per 1 ml of TRIZOL Reagent used for the initial homogenization. Mix the sample by vortexing and centrifuge at 7,500 × *g* for 5 minutes at 4°C.

**5. REDISSOLVING THE RNA**

At the end of the procedure, air-dry the RNA pellet for 5-10 minutes. Do not dry the RNA by centrifugation under vacuum. It is important not to let the RNA pellet dry completely as this will greatly decrease its solubility. Partially dissolved RNA samples have an A260/280 ratio < 1.6. Dissolve RNA in RNase-free water by passing the solution a few times through a pipette tip, and incubating for 10 minutes at 55 to 60°C. RNA can be stored at -70°C.

**ISOLATION OF SMALL QUANTITY RNA**

Isolation of RNA from small quantities of tissue (1 to 10 mg) or Cell (10^2^ to 10^4^) Samples: Add 800 µl of TRIZOL to the tissue or cells. Following sample lysis, add chloroform and proceed with the phase separation as described in step 2. Prior to precipitating the RNA with isopropyl alcohol, add 5-10g RNase-free glycogen as carrier to the aqueous phase. To reduce viscosity, shear the genomic DNA with 2 passes through a 26 gauge needle prior to chloroform addition. The glycogen remains in the aqueous phase and is co-precipitated with the RNA. It does not inhibit first-strand synthesis at concentrations up to 4 mg/ml and does not inhibit PCR.

# Removal of Contaminating DNA from RNA Preparations

1.Prepare 100µl of DNase I solution for each sample :

| RNA | 10~20µg |
| --- | --- |
| 10X DNase I Buffer | 10µl |
| DNase I | 5µl |
| RNase Inhibitor | 1µl |
| ddH_2_O | Up to 100µl |
| Total Volume | 100µl |

2. Incubate at 37℃ for 30 minutes.

# RNA cleanup

Reagent:

RNeasy® MinElute™ Cleanup Kit （Qiagen）

Ethanol (80% and 96–100%)

1. Adjust sample to a volume of 200 µl with RNase-free water. Add 700 µl Buffer RLT, and mix thoroughly.

*If starting with an RNA pellet, be sure that the pellet is dissolved in the RNase-free water (supplied) before adding Buffer RLT.*

2. Add500 µl of 96–100% ethanol to the diluted RNA, and mix thoroughly by pipetting. Do not centrifuge. Continue immediately with step 3.

3. Apply 700 µl of the sample to an RNeasy MinElute Spin Column in a 2 ml collection tube (supplied). Close the tube gently, and centrifuge for 15 s at ≥8000 x g(≥10,000 rpm). Discard the flow-through.*

*For samples >700 µl, apply the remaining sample (up to 700 µl) and repeat the centrifugation. Discard the flow-through and collection tube.**

4. Transfer the spin column into a new 2 ml collection tube (supplied). Pipet 500 µl Buffer RPE onto the spin column. Close the tube gently, and centrifuge for 15 s at ≥8000 x g (≥10,000 rpm) to wash the column. Discard the flow-through.Reuse the collection tube in step 5.

*Note: Buffer RPE is supplied as a concentrate. Before using for the first time, add 4 volumes of ethanol (96–100%), as indicated on the bottle, to obtain a working solution.*

5. Add 500 µl of 80% ethanol to the RNeasy MinElute Spin Column. Close the tube gently, and centrifuge for 2 min at ≥8000 x g (≥10,000 rpm) to dry the silica-gel membrane. Discard the flow-through and collection tube.

*Note: Following the centrifugation, remove the RNeasy MinElute Spin Column from the collection tube carefully so the column does not come into contact with the flow-through as this will result in carryover of ethanol.*

6. Transfer the RNeasy MinElute Spin Column into a new 2 ml collection tube (supplied).

Open the cap of the spin column, and centrifuge in a microcentrifuge at full speed for 5 min. Discard the flow-through and collection tube.

*It is important to dry the silica-gel membrane since residual ethanol may interfere with downstream reactions. Centrifuging with the caps open ensures that no ethanol is carried over during elution.*

7. To elute, transfer the spin column to a new 1.5 ml collection tube (supplied). Pipet 14 µl RNase-free water directly onto the center of the silica-gel membrane. Close the tube gently, and centrifuge for 1 min at maximum speed to elute.

*Smaller volumes of RNase-free water can be used to obtain a higher total RNA concentration, but this will reduce the overall yield. The yield will be approximately 20% less when using 10 µl RNase-free water for elution. Elution with less than 10 µl may not be enough to sufficiently hydrate the silica-gel membrane and is therefore not recommended.*

The dead volume of the RNeasy MinElute Spin Column is 2 µl; elution with 14 µl of RNase-free water results in an eluate with a volume of 12 µl.

**ASSESING RNA YIELD AND QUALITY**

Reagent:

TE: 10 mM Tris-HCl pH 8, 1 mM EDTA

0.4M MOPS, pH 7.0

0.1M sodium acetate

0.01M EDTA

Formaldehyde

Formaldehyde Load Dye (ambion,)

Ethidium bromide

Agarose

**1.UV ABSORBANCE**

The concentration and purity of RNA can be determined by the NanoDrop® ND-1000, be sure to zero the spectrophotometer with the DEPC Water used for sample suspension:

a) With the sampling arm open, pipette 1ul DEPC Water water or RNA sample without dilution onto the lower measurement pedestal.
b) Close the sampling arm and initiate a spectral measurement using the operating software on the PC. The sample column is automatically drawn between the upper and lower measurement pedestals and the spectral measurement made. The data is logged in an archive file on the PC.
c) When the measurement is complete, open the sampling arm and wipe the sample from both the upper and lower pedestals using a soft laboratory wipe.
d) Result( the archive files in EXCEL and JPEG file is attached):

**Concentration**

An A260 of 1 is equivalent to 40 ng RNA/ul. The concentration of RNA is therefore calculated by multiplying the A260 X 40 ng/ul. Following is a typical example: RNA is suspended in 20 µl DEPC Water ，and then 1ul RNA is used for measurement, A260 = 65.003:

RNA concentration = 65.003 X 40 ng/ul =2600.12 ng/ul

Since there are only 19 µl of the prep left after sacrificing 1 µl to measure the concentration, the total amount of remaining RNA is: 19 µl X 2600.12 ng/ul = 49.4 µg

**Purity**

The ratio of A260 to A280 values is a measure of RNA purity, and it should fall in the range of 1.8 to 2.1. Even if an RNA prep has an A260:A280 ratio outside of this range, it may function well in common applications such as Northern blotting, RT-PCR, and RNase protection assays.

**2.DENATURING AGAROSE GEL ELECTROPHORESIS**

a. Prepare the gel

Heat 1 g agarose in 72 ml water until dissolved, then cool to 60°C. Add 10 ml 10X MOPS running buffer, and 18 ml 37% formaldehyde (12.3 M).

| **10X MOPS running buffer** | |
| --- | --- |
| **Concentration** | **Component** |
| 0.4M | MOPS, pH 7.0 |
| 0.1M | sodium acetate |
| 0.01M | EDTA |

Pour the gel and allow it to set. The wells should be large enough to accommodate at least 25 µl.

Remove the comb, and place the gel in the gel tank. Add enough 1X MOPS running buffer to cover the gel by a few millimeters.

b. Prepare the RNA sample

To 3 µg RNA, add 3X volumes Formaldehyde Load Dye. Ethidium bromide can be added to the Formaldehyde Load Dye at a final concentration of 10 µg/ml. Heat denature samples at 65–70°C for 15 min.

c. Electrophoresis

Load the gel and electrophorese at 5–6 V/cm until the bromophenol blue (the faster-migrating dye) has migrated at least 2–3 cm into the gel.

d. Visualize the gel on a UV transilluminator.

The 28S and 18S ribosomal RNA bands should be fairly sharp, intense bands (size is dependent on the organism from which the RNA was obtained). The intensity of the upper band should be about twice that of the lower band. Smaller, more diffuse bands representing low molecular weight RNAs (tRNA and 5S ribosomal RNA) may be present. It is normal to see a diffuse smear of ethidium bromide staining material migrating between the 18S and 28S ribosomal bands, probably comprised of mRNA and other heterogeneous RNA species. DNA contamination of the RNA preparation (if present) will be evident as a high molecular weight smear or band migrating above the 28S ribosomal RNA band. Degradation of the RNA will be reflected by smearing of ribosomal RNA bands.

Table: RNA yield and quality

Before DNase I treated

| Sample | OD_260_ | OD_280_ | OD_260_/ OD_280_ | RNA concentratino（ng/ul） |
| --- | --- | --- | --- | --- |
| Y007NC2(NC/CV) | 34.510 | 16.860 | 2.05 | 1380.38 |
| Y6472(KD) | 36.419 | 17.992 | 2.02 | 1456.76 |

After DNase I treated

| Sample | OD_260_ | OD_280_ | OD_260_/ OD_280_ | RNA concentratino（ng/ul） |
| --- | --- | --- | --- | --- |
| Y007NC2(NC/CV) | 7.888 | 4.155 | 1.90 | 315.50 |
| Y6472(KD) | 8.265 | 4.375 | 1.89 | 330.60 |

Fig: RNA denaturing agarose gel electrophoresis

**First Strand cDNA Synthesis**

Reagents:

SuperScript. III Reverse Transcriptase (invitrogen)

RNase Inhibitor (epicentre)

10mM dNTPs Mix

oligo(dT) _18_

1. Add the following components to a nuclease-free microcentrifuge tube:

1µl of 500ng of oligo(dT) _18_

1.5ug total RNA

1µl 10mM dNTP Mix

Sterile, distilled water to 13µl

2. Heat mixture to 65°C for 5 min. and incubate on ice for at least 1 min.

3. Collect the contents of the tube by brief centrifugation and add:

| 5 X First-Strand Buffer | 4µl |
| --- | --- |
| 0.1M DTT | 1µl |
| RNase Inhibitor | 1µl |
| SuperScript III RT | 1µl |

4. Mix by pipetting gently up and down.

5. Incubate at 50°C for 60 min.

6. Inactivate the reaction by heating at 70°C for 15 min.

7. Add 91 µl of ddH_2_O to each 20µl of cDNA synthesis reaction. Mix well.

Hold the finished First Strand cDNA Synthesis Reaction on ice until the next step or store overnight at -20 °C.

**Performing Real-Time PCR**

Reagents:

2 X SuperArray PCR master mix

1. Sample Preparation:

Mix the following components in a 5-ml tube or a multi-channel reservoir:

| 2X SuperArray PCR master mix | 1050µl |
| --- | --- |
| Diluted first strand cDNA synthesis reaction | 105µl |
| ddH_2_O | 945µl |
| Total Volume | 2100µl |

2. Loading for the 96-Well PCR Arrays:

a. CAREFULLY remove the plate seal from the PCR Array.

b. Add the cocktails to the PCR Array:

c. CAREFULLY but tightly seal the 96-Well PCR Array with the optical adhesive cover.

*NOTE: Be sure that no bubbles appear in any of the wells of the PCR Array. To remove bubbles, tap the plate gently on the bench top or, if possible, centrifuge the plate briefly.*

d. Place the plate on ice while setting up the PCR program described in “Performing Real-Time PCR Detection” below.

3. Performing Real-Time PCR Detection:

| Process step | Cycles Duration Temperature Settings |
| --- | --- |
| Polymerase Activation/Denaturation | 95°C, 10 min |
| 40 amplification cycles | 95°C, 15 s |
|  | 60°C, 1 min |
|  | Optical read |
| Melting curve analysis |  |

**Analyze data: ΔΔCt Method**

1. Calculate the ΔCt for each pathway-focused gene in each treatment group.

ΔCt (group 1) = average Ct – average of HK genes’ Ct for group 1 array

ΔCt (group 2) = average Ct – average of HK genes’ Ct for group 2 array

**NOTE:** *The expression level of the housekeeping genes chosen for normalization in the* ΔΔ*Ct method must not be influenced by your experimental conditions. If one or more such genes have been previously identified by independent means and if the PCR Array reproduces those results, use the average of their Ct values in the equation above. If an appropriate housekeeping gene has not been previously identified, use the average Ct value of all five housekeeping genes, but only if the difference in the average values between the two groups to be compared is less than one (1) cycle. Otherwise, simply use zero (0) in the place of the* average of HK genes’ Ct *for each group to be compared,and rely on the consistency in the quantity and quality of your original input total RNA across your groups to effectively normalize your results.*

2. Calculate the ΔΔCt for each gene across two PCR Arrays (or groups).

ΔΔCt = ΔCt (group 2) - ΔCt (group 1)

Where group 1 is the control and group 2 is the experimental

3. Calculate the fold-change for each gene from group 1 to group 2 as 2^-ΔΔCt^.

**OPTIONAL:** *If the fold-change is greater than 1, then the result may be reported as a fold up-regulation. If the fold-change is less than 1, then the negative inverse of the result may be reported as a fold down-regulation.*
