# Supplementary material for: Analysis of lncRNA Expression in Patients With Eosinophilic and Neutrophilic Asthma Focusing on LNC_000127
Source: Front Genet. 2019 Mar 19;10:141. doi: 10.3389/fgene.2019.00141 (PMC6433975; doi:10.3389/fgene.2019.00141)
Supplement: Supplementary Text 1 — Text that presents detailed information on data analysis. [file Data_Sheet_1.zip › Supplementary Text3-sequence of LNC_000127.doc]

GCCTCAGATGGGCATGCACACGACTTCGTAAACACACTGCATGTGCTCAATTCCCAAGGTTAAGAAGAGCACTGCACATGCAGGCAGCCCACCCTAAGGGAAGAATCCTGGGAAAGAGATCAGCCTATCAAGCTCTGGGATCAAGGTTAAAGGCTCTTTTTCTCTCTTTGATCTTCAGGTGCCCACTTGGATTTCTTTTAAGAGTTCTTTCCTTTCTTTCCTCTTCTAAAGCCTTTTTAATAAACTTCCCCTTCTGCTCTGGAACTTGCCTCAGTCTCTTTTTCTGCTTTATGCCCCTCAGTCAAATTCTTTCTTCTGAGAAGACAAGGGCTGAAGTTGCTGCAGACCCCCATGAATATGCTATCAGTAACTCAGGGTAACTCAGATCTCTTCCACCACTAACAGAAATACCACGAAGTGAGTCTGAATTATTACTAACTATATTAGTTTCTTGTTGCCACTGTAACAAATTACCACAAAGTTAGTAGCTTAAAAGAACACAAATGTAATCTCTTAGTTCTAGAGGGAGGTCAAGGTGTTGAGTCCTCCTGGGGACTCTAGAGGAAAATCCATTTCCTCACCATTTTCAGCCCTTAGAGGCCCCTGCACTCCTTGATCATGGCCCCTTCCTGGGATCACTTCCACCTCTTGCTTCATTATCATGTCTCCTACTCCTCTCTGACTTCTCCTACGTCCTTCATATAAATACCATTGTGATTTCATTGTGCCCCCTTGAGTAATGCAGGAGAATCCCCTCATCTCAAGATCCTTGACTAGGACACAATTGCAAAGTTTCTTTTACCATACAAGGAAATATTCAGAGGTTTTGGAGGTCAGGATGTGGGCATATCTAAGGAGCATCATTCAGCCTACCAAAGAAACTGTAGTGGTCAATGATGGAGAACTAGGGAATGGAGTTAAAAAAAAAAAAAAAAGAACTTATTTTTGGTAGCTTCACTTATTTCTTAATATGTGAACAGATAGCAAGGAATATAGTGTATGAGATTCAGAGTTATACAGACCTGCATTCAAATCCCAGCTATGCCACTTACTAACTGTTTGACCTTAGGCAAATAACATAAACTCTCTGAGTTTCTGCTTCCTCATGGGTAAAATGAGAATAATAATATGGCTAGTATATGTGCACATGCATTATGTATTTGTTTTTAATTAAATGGGATGTACAATACATTTACAACTTTTTTTCACTCACTATTGTGTTGTTCAAGATTTGTCCATCCAGCATGCTAGTTATCCACTCTGTAGTGATGGCCATCTAGGATGCTTCAATAACCCCTGGCAAAGATGCATATGCATGTGTGTGTCCCTGTACAGGACTTGGAAACAATTGCTCTGGCATAAACATCCAGTAGTTGAAAGTACAGTTATGGGATAAGTATTGCCAGATTGCTCTCTAAAAAGAATACACCATTTCTTCTTTCACCGGAGAAACAAATGTTCTTTTTCAGCATCTTTTTGCTGACACTTAATGTTTTCTGACTCTACTTTTTCATATGATGGGTACAAAGTGGTGTCAGACTTGTTTTTTAATAGTAGTAGG

LNC_000127：

| chr1 | 89354396 | 89356080 | + |  | 1561bp |
| --- | --- | --- | --- | --- | --- |
